# Safety profile of autologous hematopoietic stem cell mobilization and transplantation in patients with systemic sclerosis

**DOI:** 10.1007/s10067-017-3954-5

**Published:** 2017-12-18

**Authors:** Grzegorz Helbig, Małgorzata Widuchowska, Anna Koclęga, Anna Kopińska, Magdalena Kopeć-Mędrek, Władysław B. Gaweł, Adrianna Spałek, Jakub Żak, Iwona Grygoruk-Wiśniowska, Robert Liwoch, Eugeniusz Kucharz, Mirosław Markiewicz

**Affiliations:** 1 School of Medicine in Katowice, Department of Hematology and Bone Marrow Transplantation, Medical University of Silesia, Dąbrowski street 25, 40-032 Katowice, Poland; 2 School of Medicine in Katowice, Department of Rheumatology, Medical University of Silesia, Katowice, Poland; 3 Students’ Research Group, School of Medicine in Katowice, Department of Hematology and Bone Marrow Transplantation, Medical University of Silesia, Katowice, Poland

**Keywords:** Autologous hematopoietic stem cell transplantation, Complications, Mobilization, Systemic sclerosis, Toxicity

## Abstract

Autologous hematopoietic stem cell transplantation (AHSCT) is thought to be effective therapeutic approach in patients with poor prognosis systemic sclerosis; however, the toxicity remains a challenge. Between years 2003 and 2016, we enrolled 18 patients with systemic sclerosis at median age at transplant of 52 years (range 24–68). The median duration of disease before AHSCT was 14 months (range 2–85). Peripheral blood stem cells were mobilized with cyclophosphamide (CY) and granulocyte colony-stimulating factor. Conditioning regimen included CY (200 mg/kg) and alemtuzumab (median dose, 60 mg) [*n* = 11], melphalan (MEL; 140 mg/m2) and alemtuzumab [*n* = 2], CY and rabbit anti-thymocyte globulin (rATG; 7.5 mg/kg) [*n* = 4], and CY alone (*n* = 1). Four deaths occurred early after transplant. There were three males and one female at median age at death of 51 years (range 24–68). The AHSCT-related deaths have been observed on days + 1, + 4, + 9, and + 15 after procedure. The causes of death included bilateral pneumonia followed by multi-organ failure in three patients and myocardial infarction in one. Three patients expired late during post-transplant follow-up, after 5, 21, and 42 months. The causes of death were disease progression in two patients and sudden heart attack in one. Eleven patients are alive after median follow-up after AHSCT of 42.0 months (range 0–95). Before proceeding to AHSCT in systemic sclerosis, there is a strong need to optimize patient selection to reduce toxicity. The administration of alemtuzumab should be avoided due to high risk of life-threatening infectious complications.

## Introduction

Systemic sclerosis is a chronic, multisystem autoimmune disease which heterogeneous manifestation results from inflammation, vasculopathy, and fibrosis. Disease outcome is variable; however, patients with diffuse cutaneous and visceral organ involvement have the worst prognosis with a significantly reduced life-span [1, 2]. It was demonstrated that rapid skin thickness progression in systemic sclerosis was associated with early renal involvement and predicted shorter survival [3]. Similarly, diffuse cutaneous involvement with any organ damage (especially lung) was found to have poor outcome [4]. Treatment approach for patients with systemic sclerosis is complex and takes into account disease stage and organ complications. Immunosuppressants remain a cornerstone of treatment, although the supportive care plays a significant role [5]. Autologous hematopoietic stem cell transplantation (AHSCT) is thought to reset the patient’s abnormal immune system and this procedure was demonstrated to be more effective if compared with intravenous pulses of cyclophosphamide in patients with poor prognosis disease. Nevertheless, the safety of AHSCT remains a challenge [6].

The aim of our study was to assess the toxicity of mobilization and transplantation in 18 patients with poor prognosis systemic sclerosis.

## Material and methods

Between years 2003 and 2016, 18 patients were enrolled in the study at the Department of Hematology and Bone Marrow Transplantation in Katowice, Poland. They were recruited after a detailed work-up performed in a close cooperation with the Department of Rheumatology in Katowice, Poland. The institutional review board approved the study design and all patients provided an informed consent in accordance with the Declaration of Helsinki.

The main indication for transplant was progressive systemic sclerosis with poor response to or no response to conventional treatment. Patients were eligible for study participation if they met the following criteria: (1) a Karnofsky score of 80 or more, (2) 70 years of age or less, (3) disease duration of < 10 years with no severe co-morbidities, (4) diffuse systemic sclerosis with the modified Rodnan skin score (mRss) ≥ 15 or less, but coexistent pulmonary involvement, and (5) early pulmonary involvement with forced vital capacity (FVC) or hemoglobin-adjusted diffusion capacity of the lung for carbon monoxide (DLCO) between 80–40% predicted or decline in FVC > 10% or DLCO > 15% on serial testing. Pulmonary arterial hypertension, renal and cardiac insufficiency, and FVC/hemoglobin-adjusted DLCO < 40% predicted were the main exclusion criteria.

The following evaluations must be performed before study entry: a detailed medical history, physical examination, complete blood count with differential, biochemistry, autoantibody assessments, mRss measurements, pulmonary function tests including the measurement of forced vital capacity (FVC), and diffusion capacity of the lung for carbon monoxide (DLCO), electrocardiogram, and echocardiogram for left ventricular ejection fraction (LVEF).

The median duration of disease before AHSCT was 14 months (range 2–85). Except skin, the most common clinical involvements included lungs (77%) and heart (44%). Eighty-three percent of study patients had positive anti-nuclear antibodies. Cyclophosphamide was the most frequent agent administered for systemic sclerosis before transplant (61%).

Peripheral blood stem cells were mobilized with cyclophosphamide (CY; 2.0 g/m2 IV on days 1–2) followed by granulocyte colony-stimulating factor (G-CSF; 10 μg/kg) from day +5 after CY administration until apheresis. The CD34-positive cells were mobilized using Optia Spectra cell separator (Caridian BCT, Lakewood, CO, USA) and cryopreserved in liquid nitrogen at − 170 C. The minimum number of stem cells required for AHSCT was 2.0 × 10^6^ CD34+ per kg of body weight (b.w.). Conditioning regimen included CY (200 mg/kg) and alemtuzumab (median dose, 60 mg) [*n* = 11], melphalan (MEL; 140 mg/m2) and alemtuzumab [*n* = 2], CY and rabbit anti-thymocyte globulin (rATG; 7.5 mg/kg) [*n* = 4], and CY alone (*n* = 1). Supportive care after transplant included co-trimoxazole and fluconazole. Thirteen patients required post-transplant G-CSF support. Blood products were irradiated and leukocyte-depleted. Patient characteristics at diagnosis are described in Table 1.

**Table 1:** Patients characteristics at study entry

Characteristic	*N* = 18
Age at diagnosis: years (median; range)	50 (23–67)
Age at transplant: years (median; range)	51.5 (24–68)
Sex: male/female	10/8
WBC count (× 10^9^/L); median, range	9.75 (4.1–17.2)
PLT count (× 10^9^/L); median, range	323 (174–740)
HGB concentration (g/dL); median, range	12.2 (9.7–15.2)
*Serum autoantibodies*, *n* (%)	
Anti-nuclear (ANA)	18 (100)
Anti-Scl70	17 (94)
Anti-RNA polymerase (ARA)	1 (6)
GFR; median, range	125.4 (53.2–225.3)
FVC (%), median, range	81.7 (62–109)
DLCO (%), median, range	64 (40–98)
LVEF (%), median, range	55 (45–65)
BMI, median, range	22.5 (17–30.5)
mRss, median, range	21.5 (8–39)
The summed Medsger severity score (mean ± SD)	7.39 (± 3.58)
*Clinical manifestation*; *n* (%)	
Raynaud phenomenon	16 (89)
Pulmonary fibrosis	9 (50)
Pulmonary hypertension	2 (11)
Abnormal electrocardiogram	8 (44)
Gastroesophageal reflux	4 (22)
Proteinuria	1 (6)
*Prior treatment*; *n* (%)	
Cyclophosphamide	11 (61)
Corticosteroids	9 (50)
Methotrexate	5 (28)
Azathioprine	1 (6)
Mycophenolate mofetil	1 (6)
d-Penicillamine	1 (6)

*BMI* body mass index, *DLCO* diffusing capacity of lung for carbon monoxide, *GFR* glomerular filtration rate, *FVC* forced vital capacity, *HGB* hemoglobin, *LVEF* left ventricular ejection fraction, *mRss* the modified Rodnan skin score, *PLT* platelets, *WBC* white blood cell

## Results

### Mobilization data

We enrolled 18 patients with systemic sclerosis (ten male and eight female) with median age at transplant of 52 years (range 24–68). Median number of two apheresis (range 1–5) was needed to harvest a sufficient number of CD34-positive cells for transplant. The median number of mobilized CD34+ cells/kg b.w. was 3.9 × 10^6^/kg (range 2.1–13.9). There have been no life-threatening complications during mobilization and stem cell collection. One patient developed fever of unknown origin and one patient suffered from mild infection of upper respiratory tract. Median hospital stay was 14 days (range 6–24). No blood support was needed.

### Transplant data

Neutrophil recovery (absolute neutrophil count > 0.5 × 10^9^/L for three consecutive days) was achieved after median of 10 days (range 0–17). The platelet engraftment (platelet count > 20 × 10^9^/L for three consecutive days without transfusions) was achieved after median of 5 days (range 0–16). Thirteen and nine patients received post-transplant red blood cells (RBCs) and platelet (PLT) support, respectively. Median number of RBCs and PLT transfusions was 2 (range 2–5) and 12 (range 2–30), respectively. Median hospital stay was 23 days (range 12–49). Transplant data were shown in Table 2.

**Table 2:** Stem cells mobilization and transplant data

Stem cells mobilization and transplant data	
CY dose (mg); median (range)	3000 (2000–7200)
G-CSF total dose (μg); median (range)	4880 (2800–12,480)
Total number of apheresis; median (range)	2 (1–5)
CD34+ cells in apheresis (%); median (range)	0.62 (0.14–1.45)
CD34+ cells in apheresis (× 10^6^); median (range)	3.9 (2.1–13.9)
Total nucleated cell count in apheresis (10^8^/kg); median (range)	8.61 (4.14–17.08)
Stem cells transplant data	
*Conditioning*	
CY; *n*	16
CY dose in mg; median (range)	12,300 (5600–18,800)
Alemtuzumab; *n*	13
Alemtuzumab dose in mg; median (range)	60 (30–60)
rATG; *n*	4
rATG dose in mg; median (range)	300 (225–375)
MEL; *n*	2
MEL dose in mg	200
*Engraftment and supportive care*	
ANC > 0.5 × 10^9^/L; day, median (range)	10 (0–17)
PLT count > 20 × 10^9^/L; day, median (range)	5 (0–16)
RBC support; units, median (range)	2 (2–5)
PLT support; units, median (range)	12 (2–30)
IVIG (g); median (range)	10 (6–24)
G-CSF (μg); median (range)	2400 (300–3600)
*Follow-up*	
Median hospital stay; (days); median, range	23 (12–47)
Follow-up duration after transplant (months), median (range)	42 (0–95)
Patients alive; *n* (%)	11 (61)
AHSCT-related death; *n* (%)	4 (22)
Late post-AHSCT deaths; *n* (%)	3 (16)
Post-AHSCT disease progression; *n* (%)	5 (45)

*AHSCT* autologous hematopoietic stem cell transplantation, *ANC* absolute neutrophil count, *rATG* rabbit anti-thymocyte globulin, *CY* cyclophosphamide, *G-CSF* granulocyte colony-stimulating factor, *IVIG* immunoglobulin, *MEL* melphalan, *PLT* platelet

Four deaths occurred early after transplant. There have been three males and one female at median age at death of 51 years (range 24–68). The AHSCT-related deaths have been observed on days + 1, + 4, + 9, and + 15 after procedure. The causes of death included bilateral pneumonia followed by multi-organ failure in three patients and myocardial infarction in one. The time from diagnosis to AHSCT was 1 year in all expired patients (details were presented in Table 3).

**Table 3:** Summary of early deaths after transplant

Age (years)	Sex	Co-morbidities	Time from diagnosis to AHSCT (mo)	LVEF (%)	Conditioning	Time from AHSCT to death (days)	Cause of death
68	F	Hypertension; arrhythmia; diabetes; hypothyroidism	12	55	CY and alemtuzumab	4	Bilateral pneumonia
53	M	Hypertension	12	55	CY and rATG	9	Bilateral pneumonia
46	M	NA	11	55	CY	1	Bilateral pneumonia probably *Candida*-related
24	M	LBBB	12	55	MEL and alemtuzumab	15	Myocardial infarction

*AHSCT* autologous hematopoietic stem cell transplantation, *CY* cyclophosphamide, *LBBB* left bundle branch block, *LVEF* left ventricular ejection fraction, *MEL* melphalan, *NA* not applicable, *rATG* rabbit anti-thymocyte globulin

The other early side effects of transplantation included fever of unknown origin (*n* = 8), transient cardiac arrhythmia (*n* = 6), mucositis grade 1/2 (*n* = 4), urinary tract infection (*n* = 1), and anxiety attacks (*n* = 1). All blood and urine cultures were negative. *Candida* species was demonstrated in throat culture in two patients and *Enterobacter cloacae* in one. One sputum culture was positive for *Candida glabrata*.

### Late safety data

Three patients expired during post-AHSCT follow-up, after 5, 21, and 42 months. The causes of death resulted from disease progression in two patients and sudden heart attack in one.

### Follow-up

Eleven patients are alive after median follow-up after AHSCT of 42.0 months (range 0–95). Among those patients, we have found a significant reduction in mRss at 12 months after transplant compared to baseline assessment. There was no significant change in lung function. Five out 11 living patients had disease progression and required the introduction of immunosuppressive treatment.

## Discussion

Stem cell transplantation remains an effective therapeutic approach for patients with severe systemic sclerosis; however, one should bear in mind its toxicity mainly affecting the cardiopulmonary system [6]. Several study groups have shown that AHSCT in systemic sclerosis resulted in long-term improvements in skin thickness and pulmonary function, but its efficacy may be tempered by relatively high treatment-related mortality and morbidity. The up-to-date largest randomized clinical trial (ASTIS) compared the safety of AHSCT vs 12 successive monthly intravenous cyclophosphamide in 156 patients randomly assigned to receive AHSCT (*n* = 79) and CY (*n* = 77). It was demonstrated that AHSCT was associated with increased treatment-related mortality in first year after procedure; however, its use outperformed CY pulses in long-term event-free survival. In total, eight deaths were deemed to be treatment-related in AHSCT cohort (10%) vs none in CY group. Viral infections were also commonly seen in AHSCT group (28%) which was probably due to ATG use. Importantly, two patients developed EBV-driven post-transplant lymphoproliferative disease. Of note is that two patients expired during mobilization/consolidation period (respiratory failure in both). Three of eight deaths were found to be cardiac-related. More than 60% of transplanted patients developed grade 3 or 4 adverse events, which mainly affected the respiratory and cardiovascular system [7]. These findings were in contrast with data reported by Burt et al. [8] in the first published randomized trial of AHSCT vs CY in patients with systemic sclerosis (ASSIST). The authors enrolled only 19 patients, ten of them were allocated to receive AHSCT. Of note is that no patient died during the study nor developed serious adverse events. This favorable toxicity profile was probably due to small number of included patients and short-term follow-up (2 years). The center experience may also play a role.

Whatever the preparative regimen is selected for transplantation in systemic sclerosis, it is obvious that this procedure carries a substantial risk of morbidity and mortality. Many factors may have an impact on the outcome of transplant procedure and they are as follows: regimen intensity, age of patient, disease duration, the Karnofsky index, and co-morbidities. A special attention should be directed at those co-morbidities which are thought to be sclerosis-related, e.g., decreased LVEF or pulmonary arterial hypertension (PAH) [9]. The use of CY in stem cell harvesting is a commonly accepted practice; however, the preparative regimens differ between centers. Regarding the latter issue, the compromise between efficacy and toxicity should be achieved. Based on the results of ASTIS protocol, the administration of CY at 200 mg/kg of b.w. with rATG 7.5 mg/kg b.w. seems to be a reasonable choice [7]. This protocol was recently implemented in our center, but past regimens were different with relatively wide use of anti-CD52 antibody—alemtuzumab. Due to heterogeneity of regimens used in our center, it is difficult to conclude on their impact on post-transplant mortality. However, five deaths occurred in patients who received alemtuzumab (three of five patients died due to fast-progressing pneumonia). Among our eight expired patients (five in the first year after transplant), CY combined with alemtuzumab was administered in 3/11 (27%), MEL with alemtuzumab in 2/2 (100%), CY in one (100%), and CY with rATG in 1/4 (25%). Two patients from our cohort died from cardiac failure as a consequence of myocardial infarction. Both patients received alemtuzumab in their conditioning. One of them, a 24-year-old male, was found to have left bundle branch block in electrocardiogram before transplant, the second, a 55-year-old male, had no prior history towards cardiac disease. Left ventricular function was normal throughout the study period as measured by LVEF. There was also no evidence of left ventricular dysfunction in electrocardiogram. Unfortunately, cardiac autopsy was not performed as deaths occurred in local hospitals. We did not observe viral reactivations in our patients and febrile neutropenia with negative cultures was the commonest side effect.

Based on the results presented by the North American Group [10], it is reasonable to avoid total body irradiation (TBI) as preparative regimen for transplant. Treatment-related death was 24% in this study and that was the reason of protocol amendment. The other groups have shown much better results with treatment-related mortality fluctuated between 6 and 17% [8, 10, 12]. We must admit that our results with mortality rate of 27% in first year after transplant were at least discouraging, but the avoidance of alemtuzumab and better patients’ selection in terms of cardiopulmonary co-morbidities resulted in significant safety improvements in the following years.

A proper patients’ selection to transplant as well as center experience may play a role in decreasing mortality. The key point is to identify patients at risk of the development of post-transplant life-threatening complications. The screening of cardiac function using only electrocardiogram and echocardiography remains insufficient in the light of our prior experience in this patient population. The clinical utility of right and left heart catheterization as well as cardiac magnetic resonance imaging has been demonstrated in recently published studies, as these tests may identify patients with occult heart disease [11, 12]. Of note is that despite such careful heart examination, four patients died from sudden cardiac arrests during mobilization and transplantation [11]. The updated recommendations regarding cardiopulmonary assessment in patients with systemic sclerosis proceeding to AHSCT have recently been published [13]. Of note is that all patients from our cohort were negatively screened towards PAH.

Interestingly, the post-transplant outcome of non-smokers was far better if compared with ever smokers in up-to-date largest study [7]. In our study group, we identified only three smokers (16%), one of them died from disease progression late after transplant.

One should also keep in mind other factors that may have an impact on post-transplant outcome. Our suggestions include the tight control of fluid status, monitoring of electrolytes, steroids before ATG, and prophylactic use of acyclovir and co-trimoxazole. The prophylactic administration of angiotensin-converting enzyme (ACE) inhibitors seems to be controversial since the cases of sclerosis-related renal crisis were reported [14].

By offering patients a transplant, we must be aware of late consequence of this procedure, especially the development of secondary autoimmune disorders or malignancies [15].

## Conclusions

AHSCT may remain an interesting therapeutic option for poor-risk patients with systemic sclerosis; however, there is a strong need to optimize patient selection to reduce toxicity and identify those who could benefit most from this procedure. It seems reasonable to propose a pre-transplant work-up which would be focused on cardiopulmonary screening to mitigate toxic effects of AHSCT. The administration of alemtuzumab should be avoided due to high risk of developing life-threatening infectious complications.
